# His bundle pacing guided by automated intrinsic morphology matching is feasible in patients with narrow QRS complexes

**DOI:** 10.1038/s41598-022-07516-6

**Published:** 2022-03-04

**Authors:** Dirk Bastian, Caterina Gregorio, Veronica Buia, Janusch Walaschek, Harald Rittger, Laura Vitali-Serdoz

**Affiliations:** 1 Arrhythmia and Electrophysiology Division, Heart and Lung Department, Hospital Fuerth, Teaching Hospital of Friedrich-Alexander University Erlangen, Jacob-Henle-Str. 1, 90766 Fuerth, Germany; 2 Biostatistics Unit, Department of Medical Sciences, University of Trieste, Trieste, Italy; 3 MOX – Department of Mathematics, Polytechnic Milano, Milan, Italy

**Keywords:** Cardiac device therapy, Arrhythmias, Biomedical engineering

## Abstract

Pace mapping and visual comparison of the local pacing response with the intrinsic QRS morphology form the mainstay of His bundle pacing (HBP). We evaluated the performance of a surface lead morphology match algorithm for automated classification of the pacing response in patients with narrow intrinsic QRS undergoing electroanatomic mapping (EAM)-guided HBP. HBP was attempted in 43 patients. In 28 cases with narrow QRS, the EnSite AutoMap Module was used for automated assessment of the QRS morphology resulting from pace mapping in the His cloud area with either a diagnostic catheter or the His lead. An intrinsic morphology match score (IMS) was calculated for 1.546 QRS complexes and assessed regarding its accuracy and performance in classifying the individual pacing response as either selective HBP (S-HBP), nonselective HBP (NS-HBP) or right ventricular stimulation. Automated morphology comparison of 354 intrinsic beats with the individual reference determined a test accuracy of 99% (95% CI 98.96–99.04) and a precision of 97.99–99.5%. For His-lead stimulation, an IMS ≥ 89% identified S-HBP with a sensitivity, specificity and positive predictive value of 1.00 (0.99, 1.00) and a negative predictive value of 0.99 (0.98, 1.00). An IMS between 78 and < 89% indicated NS-HBP with a sensitivity and specificity of 1.00 (0.99, 1.00) and 0.99 (0.98, 1.00), respectively. IMS represents a new automated measure for standardized individual morphology classification in patients with normal QRS undergoing EAM-guided HBP.

*Clinical trial registration*: NCT04416958.

## Introduction

Lead positioning for His bundle (HB) pacing (HBP) requires detailed mapping and exact fixation of the electrode in the anatomically variable HB area at a place with individually proven His capture. Conventionally, the His electrode is placed using a combination of fluoroscopic guidance and electrical mapping based on unipolar electrogram (EGM) recordings from the screw of the lead. Electroanatomic mapping (EAM) allows atraumatic, low-fluoroscopic evaluation of the HB area and displays the His voltage in a color-coded map as a target area for electrode positioning. The definitive lead deployment site is identified by pace mapping within the His cloud. The morphology of the paced QRS complex is compared visually with the intrinsic QRS in all 12 surface-ECG leads, and the type of pacing response is classified according to criteria that have been published by a multicenter HBP collaborative group^1^. Unfortunately, beyond QRS duration, there is currently no standardized measure for intra- and interindividual automatic assessment and comparison of the pacing response during HBP implantation.

The EnSite AutoMap Module (Abbott, St Paul, MN) with automated morphology matching capability has been introduced to enhance fast EAM of premature ventricular beats and ventricular tachycardias. The algorithm automatically compares the surface lead morphology of intrinsic or paced beats with a stored template of interest, instantly calculating a morphology match score for every single QRS complex. This degree of similarity may be plotted into a three-dimensional score map to guide further treatment.

Our objective was to describe a new application of the surface lead morphology match algorithm for automatic assessment of the pacing response in patients with narrow QRS and no bundle branch block (BBB) or intraventricular conduction delay (IVCD) undergoing EAM-guided HBP. The hypothesis was that appropriate cutoff values can be identified, allowing the use of the intrinsic morphology match score (IMS) for automated differentiation between selective HBP (S-HBP), nonselective HBP (NS-HBP) and myocardial right ventricular (RV) stimulation (RVP), aiming to facilitate HBP procedures and to improve the standardization and comparability of HBP results.

## Methods

### Patients

Forty-three consecutive patients underwent an attempt at permanent HBP according to the guidelines and recommendations for physiologic pacing^2^. The study analyzed 1.546 QRS complexes obtained from 28 patients with intrinsic QRS duration (QRSd) ≤ 120 ms who were implanted for symptomatic sinus node dysfunction or impaired atrioventricular conduction. Baseline characteristics and implantation data of the entire population and the study patients are shown in Table 1. All patients provided informed consent. The study was conducted in compliance with the most recent version of the Declaration of Helsinki. The registered Pace-Conduct study (NCT04416958) was approved by the responsible ethics committee (Friedrich-Alexander University Erlangen, 145_20 Bc).

**Table 1:** Baseline characteristics and procedural data.

	Total HBP N = 43	QRSd ≤ 120 ms N = 28
Age, years	71 ± 14	70 ± 17
Female, n (%)	20 (47%)	15 (54%)
BMI, kg/m^2^	27 ± 5	27 ± 5
**Indication for HBP, n (%)**		
Sinus node dysfunction	3 (7%)	2 (7%)
AV conduction disease	25 (58%)	19 (68%)
Binodal disease	5 (12%)	3 (11%)
Cardiac resynchronization	6 (14%)	0
Permanent AF	4 (9%)	4 (14%)
**Baseline electrophysiology**		
PQ interval, ms	260 ± 82	267 ± 93
QRSd, ms	124 ± 34	101 ± 11
AH interval, ms	161 ± 78	185 ± 84
HV interval, ms	65 ± 22	58 ± 13
Structural heart disease, n (%)	28 (65%)	16 (57%)
Implant success, n (%)	41 (95.3%)	26 (92.9%)
Selective HBP, n (%)	20 (49%)	13 (50%)
- Paced QRSd, ms	101 ± 13	101 ± 14
Nonselective HBP, n (%)	21 (51%)	13 (50%)
- Paced QRSd, ms	125 ± 13	124 ± 14
Implant duration total, min	93 ± 46	93 ± 46
Implant duration His lead, min	20 ± 12	22 ± 14
Fluoroscopy time total, min	7.2 ± 4.8	6.8 ± 3.6
Fluoroscopy time His lead, min	1.9 ± 1.9	2.0 ± 1.9
ED total, mSv	0.096 ± 0.259	0.056 ± 0.053
ED His lead, mSv	0.018 ± 0.022	0.016 ± 0.018
Pacing threshold His, V @ 1.0 ms	1.14 ± 1.17	0.87 ± 0.61

*AF* atrial fibrillation, *AV* atrioventricular, *BMI* body mass index, *ED* effective dose, *HBP* His bundle pacing, *QRSd* QRS duration.

### Implantation

The conventional implantation procedure for HBP as well as the feasibility and the additional value of EAM-guiding are described in detail elsewhere^3–5^. Briefly, venous access was gained either by puncture of the axillary vein or cephalic vein cut down. A decapolar mapping catheter (Inquiry, Abbott) connected to the NavX EnSite Precision Cardiac Mapping System (Abbott) was advanced into the right atrium via the C315His guiding sheath (Medtronic, Minneapolis, MN). To limit the overall mechanical stress on the guiding sheath, the tip of the sheath was left positioned at the level of the high right atrium and diagnostic mapping was performed by manipulating the steerable catheter only. First, a detailed voltage map of the HB potentials was acquired. For pace mapping within the His area, the intrinsic QRS complex was stored as a template. Pacing was performed at places with detectable HB potentials at different energy levels with a pacing cycle length of 600 ms or adopted in patients with high rate permanent atrial fibrillation. The morphology match algorithm of the NavX system automatically compared every single paced QRS morphology with the stored template in all 12 leads of the surface ECG, instantly calculating an intrinsic morphology match score (IMS) that was displayed color coded in a three-dimensional pace map. Having obtained these two maps, the target for lead positioning was defined as the His area with the highest IMS corresponding to selective or non-selective HBP according to established conventional criteria^1,4,6^. In patients with pacemaker dependency due to infranodal disease or (scheduled) AV node ablation, the area with NS-HBP and low ventricular capture threshold was preferred for providing potential safety-backup pacing by RV septal myocardial capture from the His lead^1^. The tip of the C315His sheath was placed at the target area using the steerable diagnostic catheter as guidance. The entire maneuver was continuously tracked by the EAM system with no need for a separate guidewire. Thereafter, the diagnostic catheter was replaced by the SelectSecure 3830 HB pacing lead (Medtronic). Direct connection to the EAM allowed real-time visualization, navigation, mapping and pacing from the lead^3,5^. Again, during lead positioning, automated surface lead morphology matching was used and evaluated for rapid identification of places with either selective or NS-HBP according to the established criteria^1,6^. In cases with difficult anatomy (e.g. dilated right atrium), the C315His sheath could be replaced by a deflectable sheath (C304-69, Medtronic) for reaching a more stable lead position within the target area^4^. After lead fixation, the lead stability was assessed while pulling back the guiding sheath and measurements for lead impedance, sensing and pacing thresholds were performed. A HBP threshold of less than 2.5 V@1 ms was accepted^1^. In the case of unacceptable pacing results (HBP threshold ≥ 2.5 V@1 ms, unstable lead position, atrial capture), the lead was repositioned using the same EAM-guided approach.

In two pacing dependent patients with dilated right atrium, HBP implantation failed. Catheter mapping within the His cloud identified a small area with nonselective HB capture with high output pacing. Thereafter, we were not able to obtain a stable pacemaker lead fixation within the His area with an acceptable HB pacing capture threshold after multiple attempts. Finally, in these two patients, the lead was placed slightly more anteriorly providing septal pacing with a low pacing threshold as recommended by Dandamudi and Vijayaraman^4^.

### IMS for His lead implantation

#### AutoMap settings

The EnSite AutoMap Module with automated surface ECG morphology matching capability (Abbott) provides automatic point collection based on operator-defined settings. In principle, the EAM system collects mapping points only if the associated ECG morphology is XX% similar or higher compared with the intrinsic template QRS morphology for full or any combination of the 12 surface ECG leads. The degree of similarity is instantly calculated by the EAM system for every single mapped beat and provided as IMS. The algorithm for morphology comparison is based on Pearson’s correlation coefficient p with − 1 ≤ p ≤ + 1. For the study, the reference source was set to full 12-lead ECG for scoring and timing, and the score threshold was initially set to 0% to collect all captured paced beats for further analysis. Negative values were not plotted into a map but were used for descriptive analysis as 0% values.

Initially, a 12-lead surface ECG template of an intrinsic narrow QRS complex was frozen manually, and the reference offset was set to the onset of the QRS.

**Figure 1:**
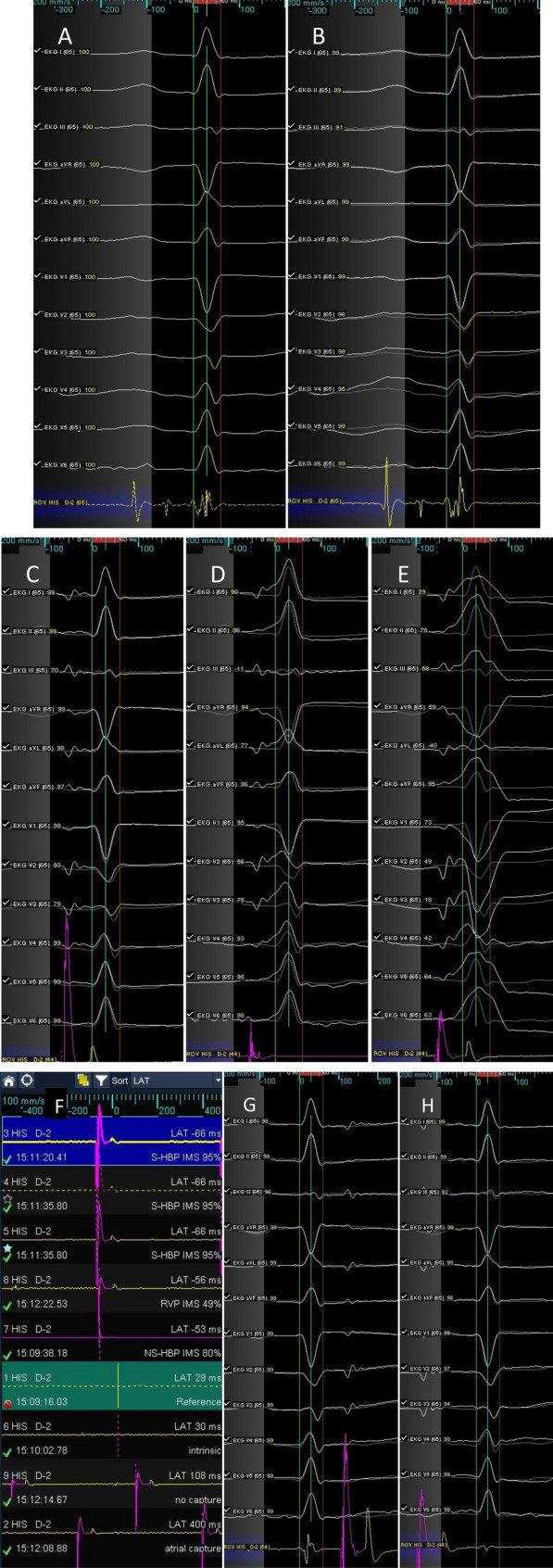
Intrinsic morphology matching. (**A**) Reference. The red ISI lines enclose the QRS morphology. (**B**) Intrinsic QRS compared with the reference. (**C**) S-HBP. The paced QRS complex (white color) is automatically compared with the stored template (gray shadow) and the degree of similarity indicated for every single lead (yellow numbers) and as IMS for all twelve leads (95%, not shown). Note that morphology comparison may be challenging for low amplitude signals, in this case in lead III. (**D**) NS-HBP, local myocardial capture, IMS 80%. (**E**) RVP, IMS 49%. (**F**) Points sorted by LAT. Positive LAT values indicate no capture or atrial capture beats. (**G**) No capture, IMS 99%, LAT positive. (**H**) Atrial capture, IMS 95%, LAT positive. 12-lead surface electrogram; ROV His, His lead.

In addition to the roving activation interval (RAI), two red independent scoring interval (ISI) lines were displayed by selecting “show scoring interval lines” and adjusted to enclose the intrinsic QRS morphology (Fig. 1A).

#### Reference evaluation

Before starting the pace map, for individual verification of the IMS algorithm accuracy and precision, a minimum of five to ten consecutive intrinsic QRS signals were compared with the stored template to ensure a high IMS match, providing a stable and reliable reference (Fig. 1B). Reference and score settings were accepted if at least 75% of the intrinsic QRS signals showed an IMS ≥ 97%.

*Pace mapping* was performed within the His cloud initially using the mapping catheter and later during His lead placement. The local pacing response was categorized by two electrophysiologists according to established criteria^1,6^, and the IMS was assessed regarding its ability to identify the type of HBP (Fig. 1C–E).

### Statistical analysis

Descriptive statistics of clinical and instrumental parameters are reported as the mean and standard deviation or as the median and interquartile range (IQR) or number and percentages for continuous variables and categorical variables, respectively. For continuous variables, comparisons between groups were performed using the nonparametric Mann–Whitney test, whereas the ${\chi }^{2}$ test or Fisher’s exact test was used for categorical variables as appropriate.

Since IMS values range between 0 and 100%, the variable was assumed to follow a beta distribution. The accuracy of the intrinsic beat reference was defined as the mode of the distribution, whereas the precision was defined as the interval between the 2.25th and 97.5th percentiles of the distribution. We refer to the Accuracy Error as 100%-Accuracy. The estimates of the mode and the 2.25th and 97.5th percentiles were derived once the beta distribution parameters (α and β) were estimated using maximum likelihood estimation. Moreover, 95% CIs were estimated using parametric bootstraps with 1000 replicates. To identify the optimum cutoffs for IMS, the optimum threshold estimation methodology for multiple classifications was used^7^. Sensibility, specificity, positive and negative predictive values (PPV, NPV) were calculated to assess the ability of the IMS to correctly classify the pacing response. A p-value < 0.05 was considered statistically significant.

### Ethics approval

The study was approved by the responsible ethics committee (Friedrich-Alexander University Erlangen, 145_20 Bc).

### Consent to participate

All patients provided written consent.

## Results

Conventional assessment and automated IMS measurement were performed and analyzed for 1,546 recorded QRS complexes in total.

### Narrow intrinsic QRS reference

**Figure 2:**
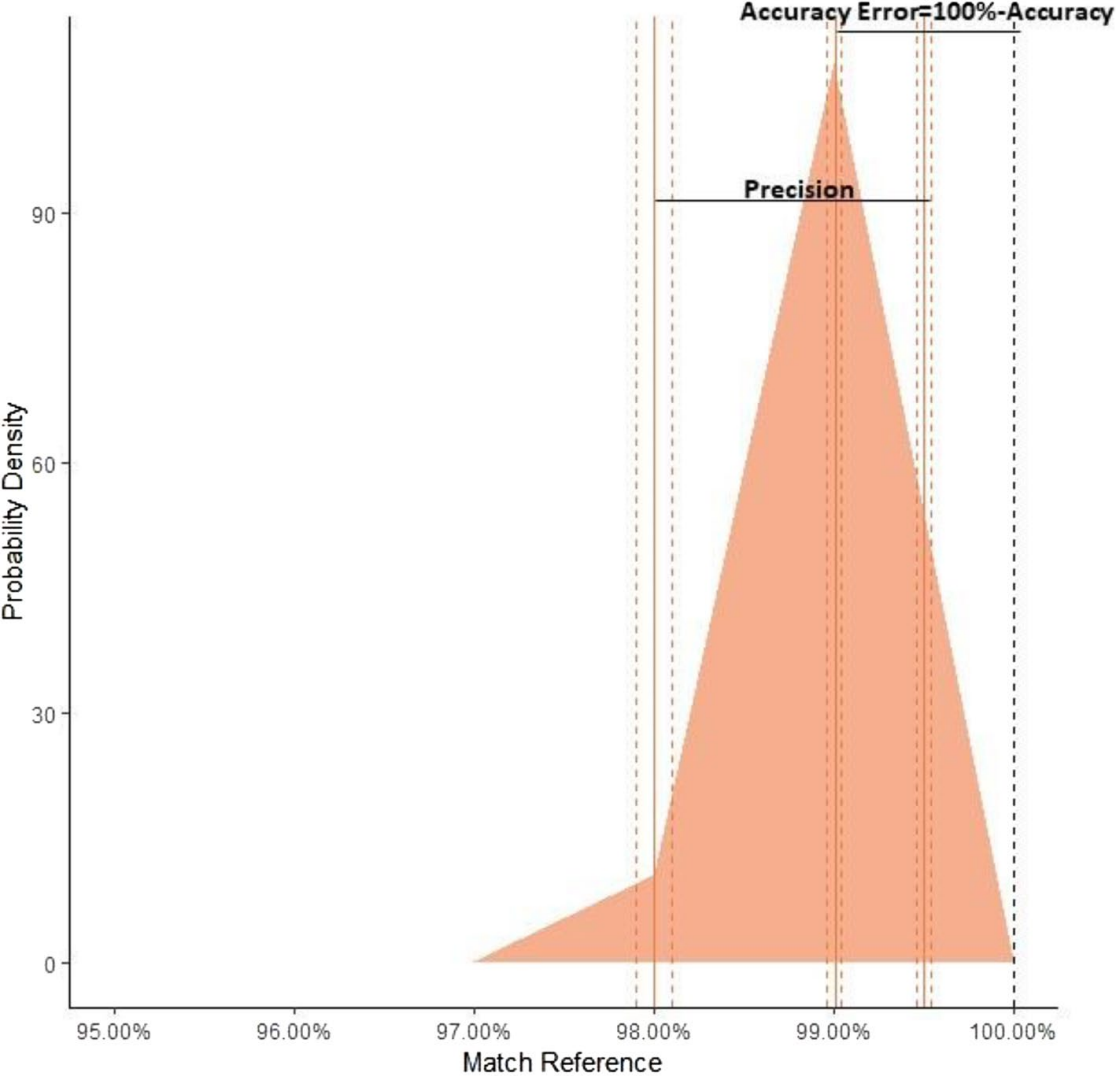
Performance of the morphology match algorithm tested by comparing 354 intrinsic narrow QRS complexes with the stored intrinsic 12-lead ECG reference. The image was created with the sotware R: The R Project for Statistical Computing (r-project.org) version 3.6.2 (R Foundation, Vienna, Austria).

Automated comparison of 354 intrinsic beats with the stored intrinsic reference resulted in a median IMS of 99% (IQR 99–100%). Taking 100% as the true value for the morphology of the individual QRS reference, the accuracy of the IMS algorithm for automated 12-lead morphology comparison was 99% (95% CI 98.96–99.04%) with a precision of 97.99% (95% CI 97.89–98.10%)–99.5% (99.45–99.54%, Fig. 2).

### Selective HBP

Five hundred sixty-three beats paced within the His area met the conventional criteria for S-HBP. Diagnostic catheter mapping resulting in S-HBP was associated with an IMS of 93% (IQR 91–96%) (Table 2). A median IMS of 93% (IQR 92–95%) was correlated with S-HBP for His lead stimulation. When comparing the IMS indicating S-HBP, no significant difference was found between pacing using the catheter and His lead stimulation (P = 0.9776).

**Table 2:** Morphology assessment for intrinsic QRS complexes and HBP using the diagnostic mapping catheter and the His lead.

	QRS morphology type	QRS N	IMS Median	IMS 1st quartile	IMS 3rd quartile	IMS min–max
Reference	Intrinsic QRS	354	99	99	100	91–100
Map catheter	Selective HBP	85	93	91	96	86–99
	Nonselective HBP	126	85	81	87	63–90
	RVP	110	23	11	46	− 7–79
His lead	Selective HBP	478	93	92	95	89–100
	Nonselective HBP	268	84	83	86	74–89
	RVP	125	48	38	58	− 9–76

Pacing response classified according to established criteria and the corresponding intrinsic morphology match score (IMS, %) as automatically calculated by the EnSite AutoMap Module.

*HBP* His bundle pacing, *RVP* right ventricular pacing.

### Nonselective HBP

In 394 cases, the pacing response was classified as NS-HBP according to established criteria. For catheter pace mapping, NS-HBP was indicated by an IMS of 85% (IQR 81–87%). Nonselective His capture by His lead pacing was associated with an IMS of 84% (IQR 83–86%).

### Myocardial RVP

With the loss of HB capture, in 235 QRS complexes, the paced morphology changed significantly, resulting in a median IMS of 23% and 48% with a large distribution for catheter and lead stimulation, respectively.

### Morphology classification by automated IMS

**Figure 3:**
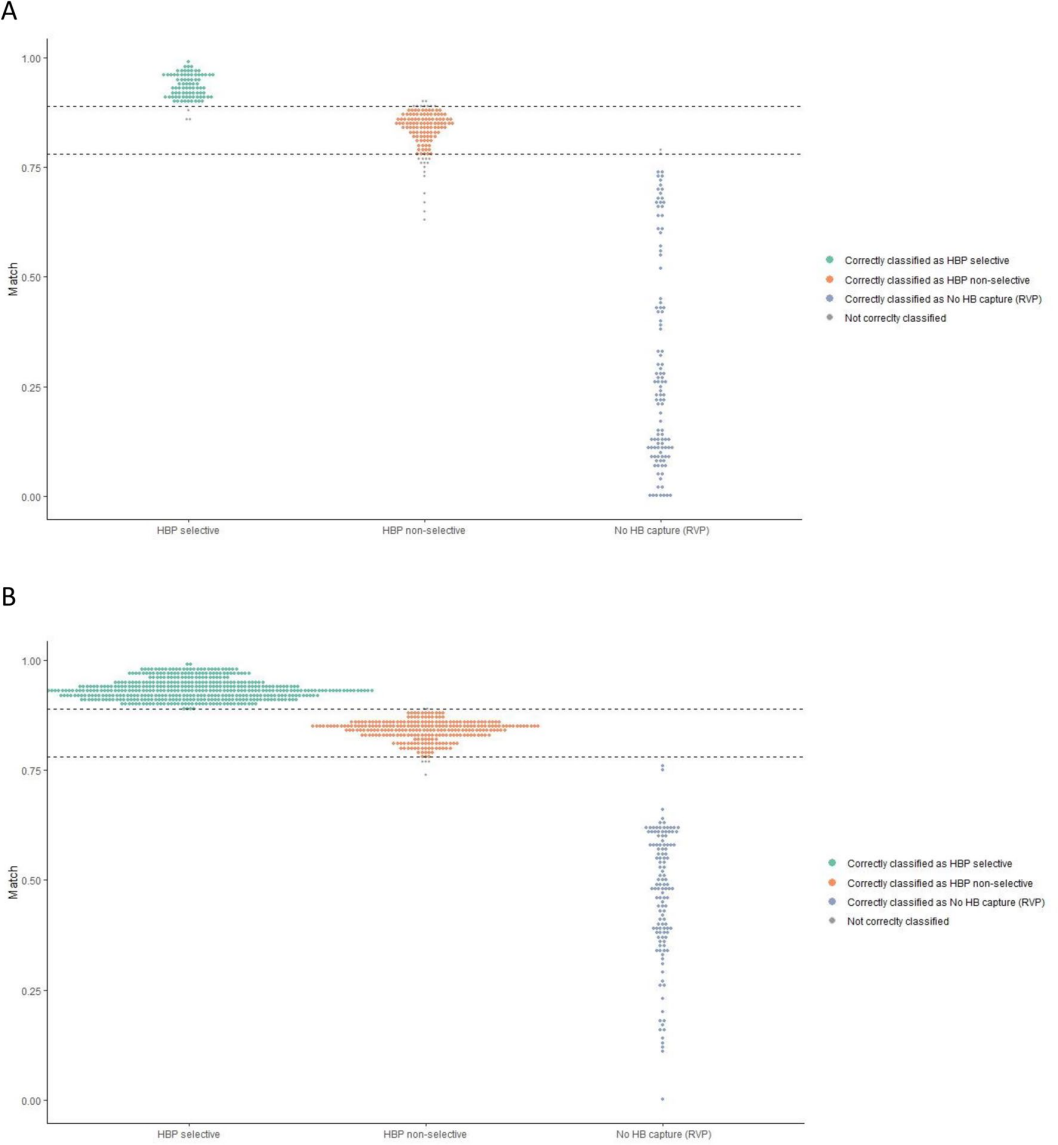
IMS-based automated classification of the pacing response. (**A**) Diagnostic mapping catheter and (**B**) His-lead pacing. IMS cutoff 0.889 for selective versus NS-HBP and 0.784 versus RVP. The image was created with the sotware R: The R Project for Statistical Computing (r-project.org) version 3.6.2 (R Foundation, Vienna, Austria).

The cutoff values best classifying the pacing response were 88.9% for differentiating selective His capture from NS-HBP and 78.4% for defining the transition to RVP (Table 3). Using these IMS cutoffs for diagnostic catheter pace mapping within the His bundle area predicted selective and nonselective HBP with sensitivities of 0.90 (0.82, 0.95) and 0.95 (0.90, 0.98) and specificities of 0.99 (0.96, 1.00) and 0.89 (0.84, 0.93), respectively (Fig. 3A).

**Table 3:** Intrinsic morphology match score (IMS)-based classification of the local pacing response.

Pacing tool	Pacing result	IMS cutoff (%)	Sensitivity	Specificity	PPV	NPV
Map catheter	S-HBP	88.9	0.90 (0.82, 0.95)	0.99 (0.96, 1.00)	0.96 (0.90, 0.99)	0.96 (0.93, 0.98)
	NS-HBP	78.4	0.95 (0.90, 0.98)	0.89 (0.84, 0.93)	0.82 (0.74, 0.88)	0.97 (0.94, 0.99)
	RVP		0.89 (0.81, 0.94)	0.99 (0.96, 1.00)	0.98 (0.94, 1.00)	0.93 (0.89, 0.96)
His lead	S-HBP	88.9	1.00 (0.99, 1.00)	1.00 (0.99, 1.00)	1.00 (0.99, 1.00)	0.99 (0.98, 1.00)
	NS-HBP	78.4	1.00 (0.99, 1.00)	0.99 (0.98, 1.00)	0.98 (0.95, 0.99)	1.00 (0.99, 1.00)
	RVP		0.97 (0.92, 0.99)	1.00 (1.00, 1.00)	1.00 (0.97, 1.00)	0.99 (0.99, 1.00)

*S-HBP* selective His bundle pacing, *NS* nonselective, *RVP* right ventricular pacing, *PPV* positive predictive value, *NPV* negative predictive value.

For pacing using the His lead, an IMS ≥ 89% identified S-HBP with a sensitivity, specificity and PPV of 1.00 (0.99, 1.00) and an NPV of 0.99 (0.98, 1.00). An IMS between 78 and 89% indicated NS-HBP with a sensitivity and specificity of 1.00 (0.99, 1.00) and 0.99 (0.98, 1.00), corresponding to a PPV and NPV of 0.98 (0.95, 0.99) and 1.00 (0.99, 1.00), respectively (Fig. 3B).

## Discussion

### Main finding

The EnSite surface-lead morphology match algorithm enables qualitative and quantitative assessment of HBP by automatic comparison of the paced QRS with an intrinsic template indicating their degree of similarity for every single ECG lead. In patients with a narrow QRS complex and no BBB/IVCD undergoing EAM-guided His lead implantation, the IMS derived from automatic 12-lead ECG morphology matching represents a simple and accurate measure for rapid and reproducible prediction and classification of the individual pacing response and a feasible tool to guide His lead positioning.

### IMS for HBP

The comparison of the paced QRS morphology with the intrinsic QRS complex in all 12 leads of the surface ECG forms the cornerstone of HBP. In patients with narrow QRS, pure His capture will produce a morphology “identical” to the intrinsic QRS – the major criterion for defining S-HBP^6^. Local myocardial capture changes the QRS morphology, which becomes wider due to a pseudodelta wave representing the morphologic criterion for NS-HBP.

At present, patient-specific morphology matching performed for every single beat during ongoing pacing remains a visual process requiring focused attention and relies on the individual expertise of the operator to recognize partially subtle differences. The lack of automation and instant quantification may prolong the time needed for individual morphology assessment and may limit further standardization as well as intra- and interindividual comparison. Recently, the diagnostic value of a programmed extrastimulus technique for the diagnosis of HB capture was shown based on the different effective refractory periods between HB and working myocardium^8^. The clinical relevance of automated tools for standardized HBP assessment was highlighted by Saini et al. by introducing a novel algorithm for device-based classification between pacing morphologies^9^.

Automated algorithms for morphology matching are well established for tachycardia discrimination in implantable defibrillator technology and have recently become integrated into modern EAM systems, aiming to facilitate mapping and ablation of premature ventricular beats and ventricular tachycardias in particular. The objective of our feasibility study was to evaluate a new application of the surface lead morphology match algorithm for automatic assessment of the pacing response in patients with narrow QRS undergoing EAM-guided implantation for HBP.

#### Morphology matching accuracy

After storing the intrinsic reference, a quick IMS assessment for five to ten consecutive intrinsic beats was used to validate the individual performance of the algorithm at the given configuration. Adequate settings and signals were provided, and the algorithm for intrinsic morphology matching was found to be highly accurate and reliable. Repetitive IMS values for the comparison of intrinsic beats with the intrinsic template < 97% should trigger a verification of signal quality and settings.

#### Catheter mapping

**Figure 4:**
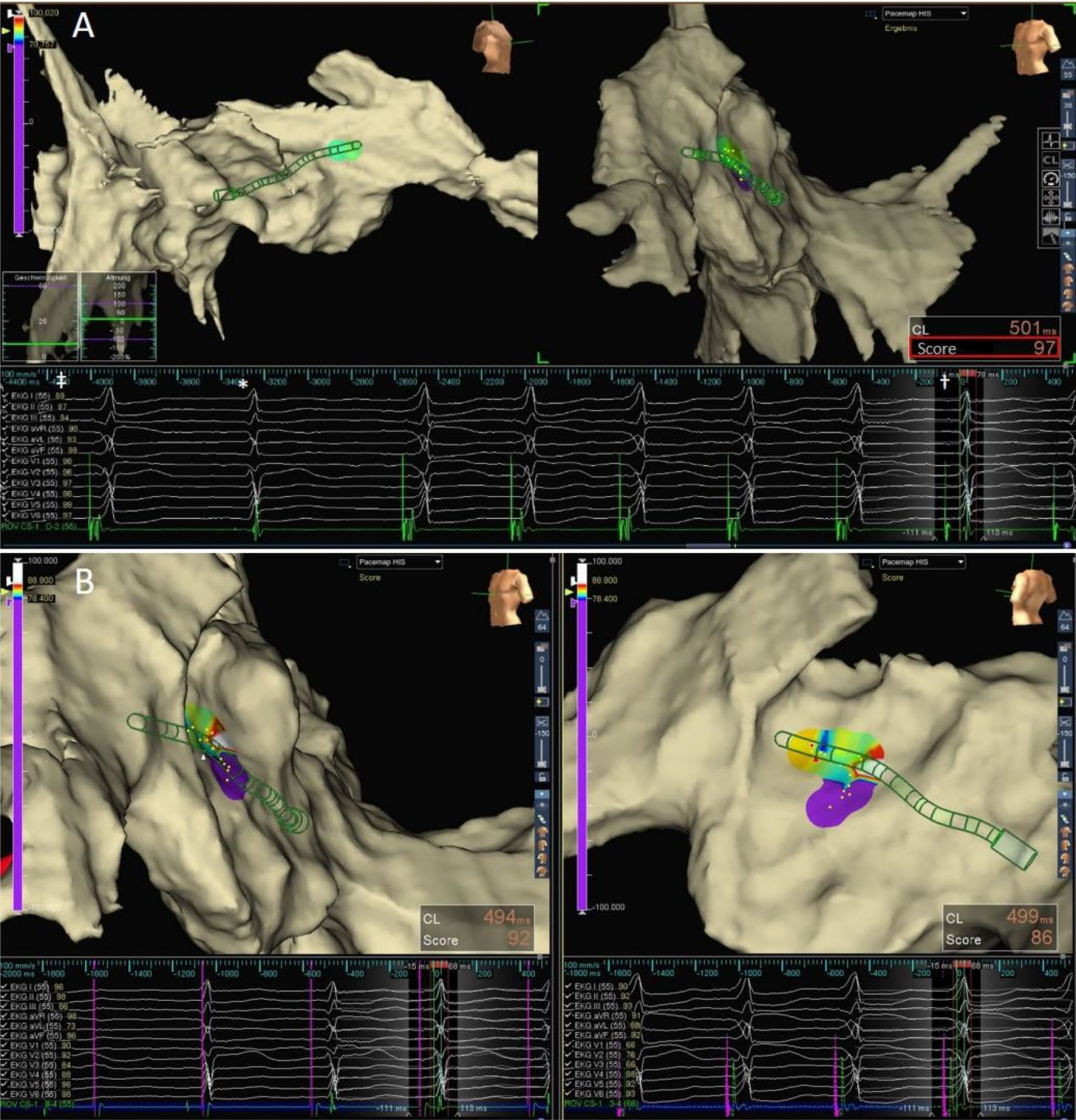
Diagnostic pace mapping. (**A**) The distal electrodes of a steerable mapping catheter were positioned at the distal His area as indicated by the second intrinsic beat (*). Local pacing with reduced pacing amplitude from 10 mA@1 ms to 5 mA resulted in the transition from NS-His capture to S-HBP (^†^). By comparing the paced morphology with the intrinsic QRS complex beat to beat the AutoMap module, the loss of local myocardial capture (S-HBP) indicated a jump in the intrinsic morphology match score from 86% (not shown) to 97% (red box). Note that in addition to the “global” IMS for the 12-lead ECG, the algorithm calculates the morphology match for every single lead (^‡^). (**B**) IMS map using the cutoff values found in the study. The ECGs show an intrinsic beat during initially ineffective pacing followed by a paced morphology with an IMS of 92%, indicating S-HBP on the left (LAO) compared with NS-HBP on the right (postero-lateral view). 12-lead surface electrogram; ROV CS, mapping catheter, distal electrodes.

Following initial identification of the His bundle area by EAM-guided voltage mapping, local bipolar catheter stimulation using automated IMS rapidly identified the area of effective HBP by automatic comparison of the paced QRS morphology with the stored intrinsic template. The resulting match scores for selective and NS-HBP were plotted into a three-dimensional IMS map serving as a target for His lead fixation (Fig. 4).

By comparing the conventionally classified pacing response with the match score calculated by the EAM system, we found well-defined IMS cutoff values for differentiating the type of HBP with high sensitivity and specificity.

Using 89% as the cutoff value, catheter pace mapping predicted S-HBP with 96%. The transition from HBP to myocardial stimulation was identified with a positive NPV and NPV of 98% and 93%, respectively.

#### His lead positioning

**Figure 5:**
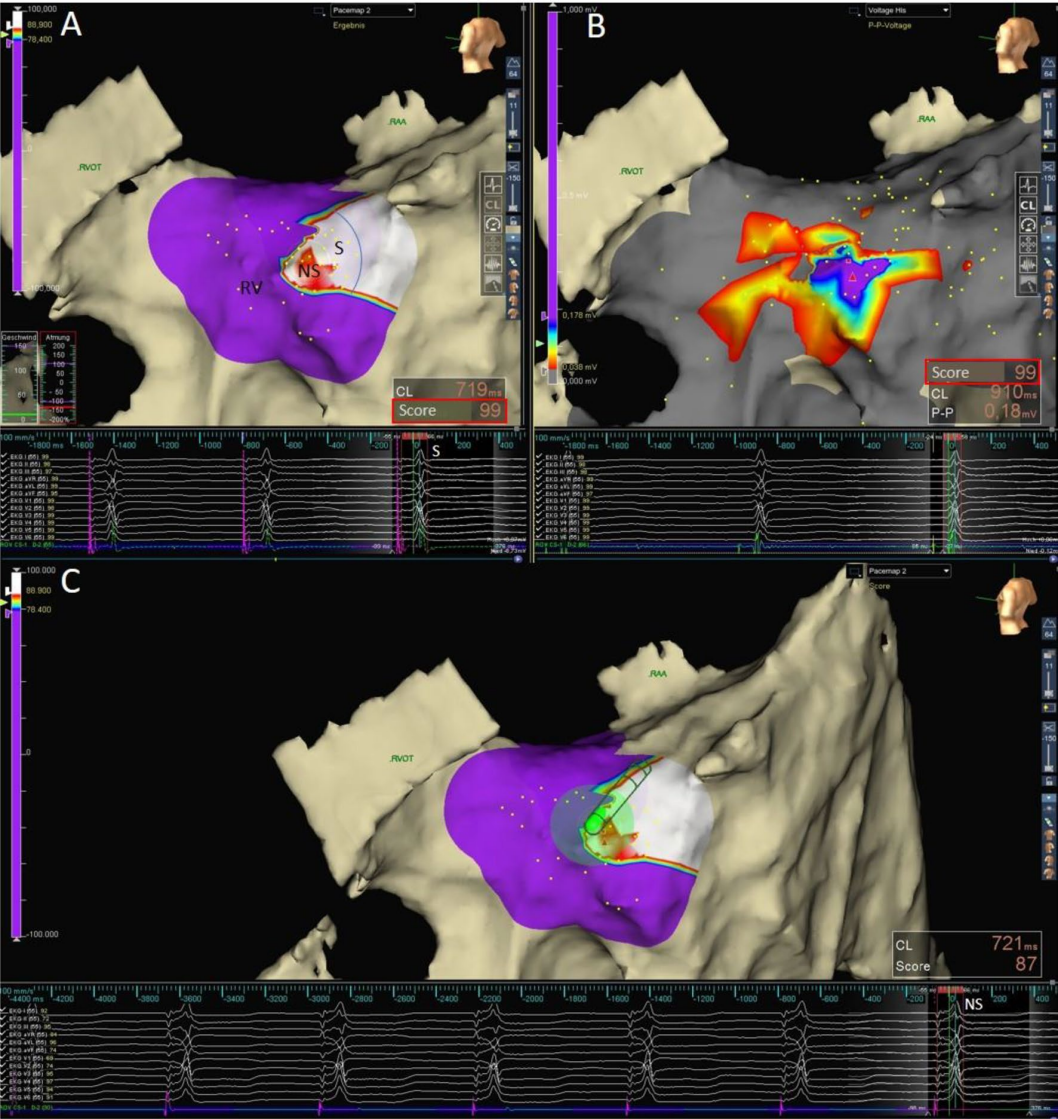
Morphology matching to guide HBP (left postero-lateral view). (**A**) Color-coded visualization of the IMS resulting from pace mapping within the His area differentiating the transition from selective (S) and NS-HBP (NS) to myocardial septal (RV) stimulation. The ECG below shows S-HBP classified by an IMS of 99% (red box). (**B**) Voltage map displaying the local bipolar voltage of the His bundle and the proximal RV conduction system peak to peak. Note the IMS of 99% for reference validation. (**C**) Fixed 3830 Select Secure lead connected to the EAM system, NS-HBP, IMS 87%. 12-lead electrogram; ROV CS, distal electrodes of the mapping catheter (**A** and **B**) and the His lead in (**C**).

Targeting the area with IMS-predicted effective HBP, automated morphology matching was further used for instant pacing response assessment during EAM-guided His lead positioning (Fig. 5). Most likely due to the more stable and punctual contact, the performance of morphology matching for lead pacing assessment was even better than that of diagnostic catheter stimulation.

#### IMS settings

The major advantage of the IMS algorithm is the operator-independent automated instant morphology comparison for all 12 surface ECG leads. Careful ISI adjustment was crucial for exactly defining the portion of the templates’ 12-lead surface morphology that is compared with the morphology of the paced QRS complex to determine the IMS between the two. If the ISI is not enabled, then the 12-lead surface IMS will be determined using the entire portion of the RAI, which may lead to incorrect results. In addition to indicating the IMS for 12-lead comparison, a match score and a template overlay for every single ECG lead are displayed, allowing for fast determination of subtle morphologic differences.

In principle, the AutoMap module allows adjustment of the automatic matching by manually shifting the stored paced QRS in relation to the reference. However, when proper settings are provided, manual correction significantly affected the IMS only for RVP without influencing the differentiation from HBP.

During pace mapping within the His area, attention must be given to recognize intermittent loss of capture or atrial stimulation to avoid misinterpretation. Following point collection, the AutoMap module allows for rapid exclusion of atrial capture or noncaptured beats by sorting the mapping points by local activation time (LAT, Fig. 1F-H).

### Acute injury to His bundle

Careful manipulation with the guiding sheath and His lead is mandatory during His lead positioning to avoid acute injury of the conduction system. Acute trauma with right BBB (RBBB) was reported by Vijayaraman et al. in 21 of 358 patients (5.9%) undergoing HBP without pre-existing His-Purkinje disease^10^. Lead-induced RBBB persisted in nine patients (2.5%) and was normalized by HBP in eight of them^10^.

In the case of transient or corrected RBBB, the initial intrinsic reference and IMS can be used further without modification, which was the case in one of our patients. It is only in rare cases with persistent BBB not normalized by HBP that the initial reference cannot be used for further morphology matching in the way it was evaluated in our study.

### Limitations

This was a nonrandomized feasibility study presenting the initial single-center experience from a small case series. However, this is the first study evaluating a new application of the surface lead morphology match algorithm for automatic assessment of the pacing response in patients with narrow QRS undergoing EAM-guided HBP. The principle was proven by assessing a high number of QRS complexes, but the number of patients, and therefore the number of different baseline QRS morphologies, was limited. Thus, the cutoff values for morphology differentiation need further evaluation in larger collectives.

The additional costs of electroanatomic mapping must be weighed against the benefits. First studies documented the feasibility and safety of EAM guidance with a significant reduction in fluoroscopy duration and exposure^5^.

The potential benefit (e.g. minimizing procedure and fluoroscopy times) during the learning curve was confirmed by Imnadze et al. using His voltage mapping to guide lead positioning^11^. In addition, EAM allows for better visualization and an understanding of the individual anatomy that may be crucial in challenging cases e.g. with dilated atrium. Sharma et al. reported on lower HB capture thresholds for EAM-guided HBP compared with conventional fluoroscopic implantation based on more detailed mapping of the HB^5^. Regarding the identification of better HB implant sites, the IMS may be of additional value by allowing for delineating areas with selective HPB, non-selective His capture and RV pacing. This information is of particular relevance in patients with pacemaker dependency due to infranodal disease or (scheduled) AV node ablation, in which the area with NS-HBP is preferred for providing backup pacing by RV septal capture from the His lead^1,12^. In these cases, the use of IMS may help to avoid an additional permanent RV backup pacing lead allowing to implant a dual chamber pacemaker instead of a CRT device thereby compensating the additional costs. Clearly, these assumptions require evaluation in further studies.

The potential clinical benefit of selective compared to NS-HBP is a matter of current evaluation. A recent large study on 350 patients undergoing HBP for bradyarrhythmic indications documented similar outcomes after 3 months follow-up regarding a combined primary endpoint of all-cause mortality or heart failure hospitalization^12^. However, although not reaching statistical significance, there was a trend in favor to S-HBP and the secondary endpoint of all-cause mortality was significantly reduced in the S-HBP group. Consequently, the authors concluded that further clinical studies with long-term follow-up are needed to evaluate the outcome of selective versus NS-HBP^12^.

In our study, the IMS was evaluated for the EnSite AutoMap Module. Other EAM systems deliver comparable automated pattern matching algorithms such as the PASO software and the CONFIDENSE Module (CARTO, Biosense Webster Inc, Irvine, CA) or the template matching system by BardEP (Boston Scientific, North Quincy, MA). The advantages and disadvantages of different EAM systems have been discussed recently^5^. In principle, all the available algorithms should provide a performance comparable to this study, but further evaluation is needed.

Our feasibility study evaluated the performance of morphology matching for HBP in patients with no BBB/IVCD. In subjects with narrow intrinsic QRS but incomplete BBB or hemiblock, corrective HBP will be associated with a different IMS. In these cases, other strategies for (semi)automated morphology assessment may be applied and this is a topic that requires further evaluation. It is important to note that in patients with BBB/IVCD, taking the wide intrinsic QRS as a reference, a high IMS would indicate HBP without correction. Corrective HBP changes the morphology and narrows the QRS associated with a lower IMS. Therefore, the approach investigated for normal intrinsic QRS morphology is not one-to-one applicable to patients with BBB/IVCD. In these cases, the region with the narrowest paced QRS represents the target region for corrective HBP. Unfortunately, the current version of the EnSite AutoMap Module does not provide automatic tracking of the QRS duration in the surface ECG. Thus, at present, the trigger is set at the earliest QRS deflection, and the end of the surface QRS is defined manually, thereby creating a “reversed late potential” map displaying the QRSd, as shown in a recent case report^13^. This approach may be investigated in further studies, and in addition, we propose to integrate an algorithm for automated QRSd measurement into the NavX system to enable the creation of an automatic map displaying the region with the best corrective pacing in patients with BBB/IVCD. Alternatively, once corrective conductive system capture has been achieved by diagnostic pace mapping, the resulting corrected paced QRS morphology can be stored as a new reference for IMS-based mapping to guide further lead placement.

## Conclusion

Using the EnSite AutoMap Module automates the process of pacing response evaluation and classification by providing instant automatic comparison of the paced QRS morphology at any given point in the His cloud with a stored intrinsic template in the 12-lead surface ECG based on user-defined settings. The resulting degree of concordance (IMS) can be automatically plotted into a detailed 3D electroanatomic map to guide further HBP.

The IMS enables accurate and reproducible automated differentiation between S-HBP, NS-HBP and RVP in patients with normal intrinsic QRS. The standardized automated measure is largely independent of subjective visual grading, thereby allowing more precise evaluation and improved comparability of the individual pacing results. Automated assessment of HBP in patients with BBB/IVCD and a possible integration of morphology matching in electrocardiogram systems require further evaluation.

## Data Availability

The data will be shared upon reasonable request addressed to the corresponding author.

## Abbreviations

BBB: Bundle branch block
EGM: Intracardiac electrogram
EAM: Electroanatomic mapping
HB: His bundle
HBP: His bundle pacing
IMS: Automated intrinsic morphology match score
ISI: Independent Scoring Interval
IVCD: Intraventricular conduction delay
NPV: Negative predictive value
NS-HBP: Nonselective HBP
PPV: Positive predictive value
QRSd: QRS duration
RBBB: Right bundle branch block
RV: Right ventricular
RAI: Roving activation interval
RVP: RV pacing (no His bundle capture)
S-HBP: Selective HBP
